# Investigation of cationic ring-opening polymerization of 2-oxazolines in the “green” solvent dihydrolevoglucosenone

**DOI:** 10.3762/bjoc.19.21

**Published:** 2023-02-28

**Authors:** Solomiia Borova, Robert Luxenhofer

**Affiliations:** 1 Functional Polymer Materials, Chair for Advanced Materials Synthesis, Institute for Functional Materials and Biofabrication, Department of Chemistry and Pharmacy, Julius-Maximilans-University of Würzburg, Röntgenring 11, 97070 Würzburg, Germany; 2 Soft Matter Chemistry, Department of Chemistry and Helsinki Institute of Sustainability Science, Faculty of Science, University of Helsinki, PO Box 55, 00014 Helsinki, Finland

**Keywords:** 2-alkyl-2-oxazolines, matrix-assisted laser desorption/ionization mass spectrometry, nuclear magnetic resonance, polymerization kinetics

## Abstract

For about the last ten years, poly(2-oxazoline)s have attracted significant attention as potential material for biomedical applications in, e.g., drug delivery systems, tissue engineering and more. Commonly, the synthesis of poly(2-oxazoline)s involves problematic organic solvents that are not ideal from a safety and sustainability point of view. In this study, we investigated the cationic ring-opening polymerization of 2-ethyl-2-oxazoline and 2-butyl-2-oxazoline using a variety of initiators in the recently commercialized "green" solvent dihydrolevoglucosenone (DLG). Detailed ^1^H NMR spectroscopic analysis was performed to understand the influence of the temperature and concentration on the polymerization process. Size exclusion chromatography and matrix-assisted laser desorption/ionization time-of-flight mass spectrometry were performed to determine the molar mass of the resulting polymers. Our work shows clearly that the solvent is not inert under the conditions typically used for the cationic ring-opening polymerization, as evidenced by side products and limited control over the polymerization. However, we could establish that the use of the 2-ethyl-3-methyl-2-oxazolinium triflate salt as an initiator at 60 °C results in polymers with a relatively narrow molar mass distribution and a reasonable control over the polymerization process. Further work will be necessary to establish whether a living polymerization can be achieved by additional adjustments.

## Introduction

Hydrophilic synthetic polymers are interesting for a wide range of applications, including in the biomedical field. The synthesis of these polymers often requires the use of various organic solvents. However, most of the used solvents are undesirable on a large scale for health, safety, and environmental reasons [1]. Halogenated solvents such as chlorobenzene or dichloromethane are widely used in polymer production, but their toxicity and high energy consumption for synthesis make them unwanted for widespread commercial use. Many other dipolar solvents such as dimethyl sulfoxide (DMSO), *N*,*N*-dimethylformamide (DMF), *N*-methyl-2-pyrrolidone (NMP), acetonitrile (MeCN), etc., have been added to the registration, evaluation, authorization and restriction of chemicals (REACH) list because of their negative environmental impact [1–2]. Therefore, the search for suitable solvents for the synthesis of synthetic hydrophilic polymers with a good safety and environmental profile has been a hot topic in recent years. Specifically, the search for more benign solvents for the polymerization of 2-alkyl-2-oxazolines has been ongoing for some time.

Poly(2-oxazoline)s (POx) are a family of polymers investigated for a range of biomedical applications, in particular as an alternative to the hydrophilic poly(ethylene glycol) (PEG), which is the polymer of choice for many biomedical applications due to its high solubility, low cytotoxicity and biocompatibility [3–9]. However, an ongoing discussion about pre-existing antibodies [10–13], anaphylaxis [14–15], and vacuolization [12], drives the search for alternatives [4,8]. POx have attracted the attention of scientists and companies in recent years as a promising material for pharmaceutical applications [16–17].

**Figure 1:**
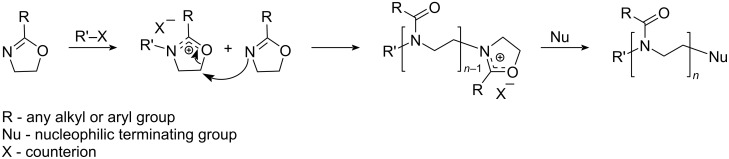
Schematic representation of the mechanism of the cationic ring-opening polymerization (CROP) of 2-alkyl-2-oxazolines.

POx are obtained via cationic ring-opening polymerization (CROP) of the respective 2-substituted 2-oxazoline (Figure 1) initiated with various electrophiles [18–21]; most commonly alkyl tosylates or triflates are applied [21–24]. The use of suitable solvents plays a significant role in obtaining well-defined polymers with a narrow molar mass distribution [25]. The question of the “right” solvent, however, has a critical influence not only on the resulting polymer but also on the environment. More than 20 million tons of waste residues from used organic solvents are emitted into the atmosphere, polluting the environment every year [26]. Therefore, choosing environmentally benign solvents becomes ever more relevant [27]. Accordingly, some research groups have been also looking for "green" and safer solvents suitable for CROP of 2-oxazolines. "Green" solvents are considered environmentally friendly, less hazardous solvents that make a product or process to have the least environmental impact throughout its life cycle [28–29]. Generally, DMF, NMP, and MeCN, or, more recently benzonitrile are solvents of choice for the 2-oxazoline polymerization and copolymerization. However, none of these are benign from a safety or environmental point of view [28]. Accordingly, Hoogenboom et al. investigated the CROP of 2-alkyl-2-oxazoline in sulfolane and investigated its effect on monomer distribution and self-assembly of the performed copolymer [30–32]. However, the degradability of sulfolane into acidic components, its relatively high melting point, and difficulties during handling are challenges for a wider application [33] along with some residual toxicity. Alternatively, Vergaelen and co-workers investigated ethyl acetate as a solvent for the CROP of 2-ethyl-2-oxazoline (EtOx) [23]. However, ethyl acetate is not a suitable solvent for most POx, accordingly, it was used successfully only for the polymerization of EtOx [34]. Correia et al. used supercritical carbon dioxide for the synthesis of 2-oxazoline-based oligomers with antimicrobial properties and applied boron trifluoride etherate as the initiator [35]. However, carbamic acid polymer termini from the initiation were observed, and apparently only syntheses of polymers with a low molar mass could be achieved. In addition, the specific equipment necessary for supercritical CO_2_ applications will limit its widespread use.

Dihydrolevoglucosenone (DLG) is a dipolar aprotic bio-based solvent obtained from cellulose [36–37], commercially known as Cyrene^TM^, and has been introduced as a new "green" solvent marketed specifically as an alternative to solvents such as DMF or NMP, which are particularly interesting for CROP of POx. It is reported that DLG is safe to handle, environmentally friendly, highly stable towards oxidation, yet degrades into water and carbon dioxide [27,38]. Therefore, DLG is an interesting candidate to investigate as solvent for the CROP of 2-oxazolines. Accordingly, in this work, we present the first examples of the successful CROP of POx and discuss the challenges associated with this approach. We found that it is critical to use suitable initiator species to exert some control over the polymerization. Nevertheless, CROP of 2-oxazolines in DLG remains plagued with some side reactions at this point, limiting the definition of the obtained polymers. Whether further optimizations can overcome these limitations remains to be seen.

## Experimental

### Materials and methods

All substances were purchased from Sigma-Aldrich (Steinheim, Germany) and were used as received unless otherwise stated. The monomer 2-*n*-butyl-2-oxazoline (BuOx) was prepared following the procedure by Witte and Seeliger [39]. All reagents for polymerization including methyl *p*-toluenesulfonate (MeOTs), methyl trifluormethanesulfonate (MeOTf), 2-methyl-2-oxazoline, 2-ethyl-2-oxazoline, and 2-butyl-2-oxazoline were refluxed under CaH_2_, distilled, and stored under an inert atmosphere. Dihydrolevoglucosenone (DLG) was obtained from Sigma-Aldrich and purified by twice fractionation distillation or via distillation over BaO under reduced pressure and stored under argon atmosphere. 2-Ethyl-3-methyl-2-oxazolinium triflate salt (EtOxMeOTf) was synthesized according to the reported procedure [40].

The kinetic study of the polymerization was monitored by ^1^H NMR spectroscopy analysis. NMR spectra were recorded on a Fourier 300 spectrometer (^1^H; 300.12 MHz and ^13^C (^1^H); 75.48 MHz; Bruker Biospin; Rheinstetten, Germany) at a temperature of 298 K and evaluated using the MestReNova V.6.0.2.-5475 software (Mestrelab Research, Santiago de Compostela, Spain).

Size exclusion chromatography (SEC) measurements were performed on a Polymer Standard Service SECurity (PSS, Mainz, Germany); precolumn: 50 × 8 mm PSS PFG linear M; 2 columns: 300 × 8 mm PSS PFG linear M (particle size 7 µm; pore size 0.1–1,000 kDa) at 313 K. HFIP was supplemented with 3 g/L potassium triflate, and the flow rate was adjusted to 0.50 mL/min. Calibration was performed using PEG standards with molar masses ranging from 0.1–1,000 kg/mol. Before every measurement, samples were filtered through 0.2 µm PTFE filters, Roth (Karlsruhe, Germany). Obtained data were processed with Win-GPC software.

Matrix-assisted laser desorption/ionization time-of-flight mass spectra (MALDI-TOF MS) were recorded on an Autoflex II (Bruker Daltonics, Bremen, Germany) using an N_2_ laser (λ = 337 nm). All spectra were recorded in positive reflector mode. Detection was typically set from 1000 *m*/*z* to 7000 *m*/*z*. After parameter optimization, the instrument was calibrated with PEG standards depending on the *m*/*z* range of the individual sample. Samples were prepared with sinapinic acid (3,5-dimethoxy-4-hydroxycinnamic acid, SA) as matrices using the dried-droplet spotting technique (0.5–1.5 µL). Samples (1 g/L) were dissolved in MeOH (supplemented with 1.0% TFA).

Thermogravimetric analysis (TGA) of the resulting polymers was run on a TG 209F1 IRIS (NETZSCH, Selb, Germany). The samples (5–10 mg) were placed into aluminum oxide crucibles (NETZSCH, Selb, Germany) and heated from 25 °C to 900 °C with a 10 K/min heating rate and the mass loss measured. The resulting data were evaluated with the NETZSCH Proteus – Thermal Analysis – V.5.2.1 software.

Dynamic scanning calorimetry (DSC) was performed on a DSC 204F1 Phoenix (NETZSCH, Selb, Germany) under a N_2_ atmosphere (20.0 mL/min). Samples were placed in aluminum pans, heated up to 200 °C and then cooled to −50 °C (10 K/min). The heating/cooling was repeated two additional times. The resulting thermograms were analyzed with the NETZSCH Proteus – Thermal Analysis – V.5.2.1 software.

The polymerization of 2-alkyl-2-oxazolines was performed as follows: 1.0 equiv of the initiator was placed into a dried and argon-flushed flask and dissolved in a respective amount of solvent. Once the monomer was added, the reaction mixture was placed into a preheated (up to the investigated temperature) oil bath and incubated till the full monomer conversion. Monomer conversion was monitored by ^1^H NMR spectroscopy. After complete monomer consumption, the reaction was terminated by adding 3.0 equiv of water and left to react overnight at 45 °C. The polymer was purified by precipitation from cold diethyl ether (Et_2_O) or direct dialysis against water overnight, followed by lyophilization. The resulting polymer was obtained as a slightly yellow powder.

The synthesis of 2-ethyl-3-methyloxazolinium triflate was carried out as follows: 30 mL of dry diethyl ether were placed in a dry round-bottomed flask and methyl trifluoromethanesulfonate (2.38 g, 14.50 mmol, 1.2 equiv) was added. The mixture was cooled in an ice bath. Then, 2-ethyl-2-oxazoline (1.20 g, 12.10 mmol, 1.0 equiv) was added slowly. A slight turbidity of the solution was observed. Over time an oily and colorless liquid phase was formed. The diethyl ether phase was separated and the remaining oil was further dried for 10 h at 1 mbar. A white powder was obtained with a moderate yield of 74% (2.37 g).

## Results and Discussion

### Polymerization of 2-methyl-2-oxazoline

The CROP of 2-oxazolines is commonly initiated by alkyl tosylates [41–43] and alkyl triflates [41–42] and to a lesser extent, alkyl halides [42,44]. Accordingly, we first investigated the CROP of MeOx in DLG with a targeted degree of polymerization (DP) of 50 and 100 using methyl trifluoromethanesulfonate, also commonly referred to as methyl triflate (MeOTf) at 60 °C, 90 °C, and 120 °C. These first experiments did not show satisfying results. The polymerization at 120 °C displayed a maximal monomer conversion of approx. 60% already after 15 min, but further incubation did not show an increase of monomer conversion. Application of 90 °C and 60 °C led to decreased monomer conversion down to ca. 32% and 16% after 75 min of incubation, respectively. However, further incubation up to 24 h also did not show a significant progress of monomer conversion. This suggests side reactions interfering with the propagating species and termination. It is important to note that the color of the polymerization mixture rapidly turned yellow, which is another sign of undesired side reactions.

Besides, ^1^H NMR spectra of the obtained polymers during the polymerization process (Figure S1 in Supporting Information File 1) demonstrate the appearance of new signals at 1.65 ppm (box a), 2.84 ppm (box b), 3.20 (box c), 3.82 ppm (marked with an asterisk), 4.20–4.75 ppm (box d) and 5.16–6.75 ppm (boxes e–g) which cannot be attributed to the desired product poly(2-methyl-2-oxazoline) (PMeOx).

We have already mentioned that MeOTf is a widely used initiator for LCROP 2-oxazolines polymerization. Due to its high reactivity, MeOTf can react with 2-oxazoline monomers or solvents well below room temperature, while propagation only proceeds at above 40 °C. The resulting triflate counterion ensures polymerization via the cationic mechanism, but the extremely high reactivity of MeOTf might be an issue when using DLG as the solvent. Therefore, we considered the use of the initiator salt, i.e., the product of the stoichiometric reaction of MeOTf and a 2-oxazoline monomer. This would remove the excessive reactivity but retain the benefit of having triflate counterions for cationic polymerization. Accordingly, 2-ethyl-3-methyl-2-oxazolinium triflate (EtOxMeOTf) was synthesized. The ^1^H NMR analysis showed all expected resonances and in particular the signals at 4.98 ppm and 4.29 ppm, attributed to the typical methylene protons in the oxazolinium ring (Figure S2, Supporting Information File 1). Unfortunately, regarding the polymerization of MeOx, there was no improvement when the initiator salt EtOxMeOTf was introduced directly. The molar mass of the resulting PMeOx remained much lower than expected with a broad molar mass distribution.

The comparison of the polymerization kinetics using MeOTf and EtOxMeOTf requires a comparison of polymerization rates. Therefore, the apparent rate of polymerization depending on the initiator used was also calculated. The apparent propagation rate (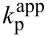) can be calculated according to the equation below, where [M]_t_ is the concentration of the monomer over time, [I]_0_ is the concentration of the initiating group (which equals the concentration of the propagation species [P*] if a fast and quantitative initiation is achieved), [M]_0_ is the initial concentration of the monomer, and *t* is the reaction time.


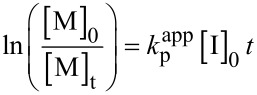


Considering that *k*_p_ is constant and [P*] should be constant and equal to [I]_0_, a linear plot of the dependence of monomer concentration M on *t* is expected in the case of the living polymerization. The 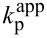 decreases compared with MeOTf at the same temperature. The data obtained from these kinetic investigations are summarized in Table 1. After examining the ^1^H NMR spectra collected during the polymerization, we suppose that a rapid termination and chain transfer reaction occurs during the MeOx polymerization. According to the chain transfer mechanism introduced by Litt et al., MeOx acts as a base and abstracts a proton from the polymer side chain [45]. Due to the slightly increased nucleophilicity, the resulting protonated monomer leads to further propagation. Sedlacek et al. also indicated a higher chain transfer reaction rate for MeOx than for EtOx during their polymerization in sulfolane [31].

**Table 1:** Investigation of the cationic ring-opening polymerization of 2-methyl-2-oxazoline initiated with methyl triflate at 60 °C, 90 °C, and 120 °C and with 2-ethyl-3-methyl-2-oxazolinium triflate salt at 90 °C in DLG.

Initiator	MeOx							

	Temperature, °C	*n*	Conv., (NMR) %	DP^a^	*M*_n_^a^, kg/mol	*M*_n_^exp^ (GPC) kg/mol	*Ð* ^b^	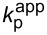 Lmol^−1^s^−1^ *10^−3^

MeOTf	120	50	62	30	3.05	n.d	n.d	n.d
	90		32	16	1.40	0.95	1.14	0.6
	60		16	8	0.73	0.51	1.12	0.2
EtOxMeOTf	90		48	25	2.16	0.37	1.49	0.3

^a^Calculated according to the monomer conversion as obtained from ^1^H NMR spectra. ^b^*Đ* = *M*_w_/*M*_n_ where *M*_w_ is the mass-average molar mass (or molecular weight) and *M*_n_ is the number-average molar mass (or molecular weight).

To summarize, the polymerization of MeOx in DLG proved to be problematic. Changes in temperature and/or initiator did not yield considerable improvements. It is known that MeOx polymerization can be challenging due to the poor solubility of PMeOx in some solvents [30–31,34]. However, no precipitation was observed during polymerization, which rules out the solubility as an issue. Therefore, we decided to investigate the polymerization of 2-alkyl-2-oxazolines with a longer side chain, as these have slightly lower reactivity. In particular, we investigated the polymerization of 2-ethyl-2-oxazoline (EtOx) and 2-butyl-2-oxazoline (BuOx).

### Incubation of dihydrolevoglucosenone with the initiator

Dihydrolevoglucosenone (DLG) is prepared by hydrogenation of levoglucosenone in the presence of palladium as a catalyst. Recently, Debsharma et al. reported the CROP of levoglucosenyl alkyl ether in CH_2_Cl_2_ at 0 °C and at room temperature using triflic acid or boron trifluoride etherate as initiators [46–48]. The ^1^H NMR spectra of the resulting polymers showed significant signals at 6.11–5.92 ppm, 5.93–5.83 ppm, 4.36–4.27 ppm, and 3.92–3.82 ppm. The prepared unsaturated polyacetal was then hydrogenated to give poly(6-ethyl-2,3-dimethoxytetrahydro-2*H*-pyran). The ^1^H NMR spectrum of the hydrogenated product showed no signals at 6.11–5.92 ppm and 5.93–5.83 ppm, but the appearance of new signals at ca. 1.45–2.00 ppm [46,48].

As a control experiment, DLG was incubated with MeOTf for 4 h at 90 °C. The resulting product was purified by precipitation from cold Et_2_O. The determination of *M*_n_ by SEC analysis was about 5.9 kg/mol with a wide molar mass distribution and the presence of a high molar mass shoulder (Figure S3 in Supporting Information File 1). The ^1^H NMR spectrum showed the presence of broad signals at 1.45–2.47 ppm, 2.55–2.75 ppm, 3.49–4.59 ppm, 4.64–4.76 ppm, and 4.84–5.45 ppm.

The same chemical shift was observed after incubation of the solvent with EtOxMeOTf at 0 °C (Figure S4a, Supporting Information File 1). The SEC traces of the product obtained after incubating DLG with the oxazolinium salt were about 2.0 kg/mol with a high molecular weight shoulder (Figure S4b, Supporting Information File 1). TGA analysis of the product showed a slight loss of mass from 25 °C to 105 °C associated with the removal of residual water, followed by a loss of 11% of the resulting mass over a temperature range of about 100 °C to 200 °C with further degradation from 236 °C to 460 °C (Figure S5a in Supporting Information File 1). The analyzed product exhibited a glass transition temperature (*T*_g_) at 140 °C, and no melting point was observed by DSC (Figure S5b, Supporting Information File 1), pointing to an amorphous nature of the resulting material. Incubation of EtOx with DLG at 90 °C showed no new signals in the NMR spectra (Figure S6), indicating no interaction between the solvent and the monomer.

### Polymerization of 2-ethyl-2-oxazoline

**Figure 2:**
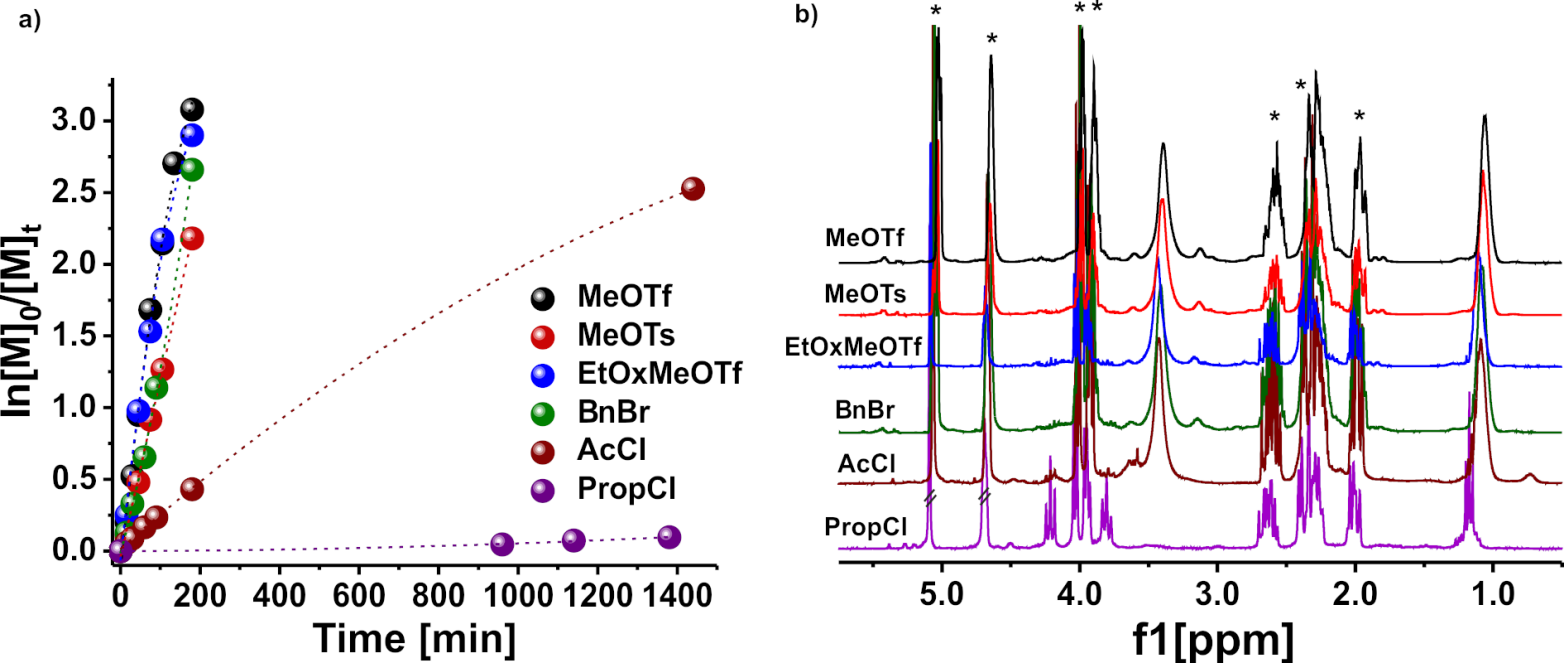
(a) First-order kinetic plot for the 2-ethyl-2-oxazoline ring-opening polymerization in DLG at 90 °C using 3 M monomer concentration and a monomer/initiator ratio of 50. The lines have been added to guide the eye. (b) ^1^H NMR (300 MHz, CDCl_3_) spectra of the 2-ethyl-2-oxazoline polymerization initiated with methyl triflate (black), methyl tosylate (red), 2-ethyl-3-methyl-2-oxazolinium triflate (blue), benzyl bromide (green) after 3 h of incubation and acyl chloride (dark red), propionyl chloride (purple) after 24 h of incubation in DLG. Peaks marked with asterisks originate from residual solvent signals.

Six different electrophiles, acetyl chloride (AcCl), propionyl chloride (PrCl), benzyl bromide (BnBr), MeOTf, MeOTs, and EtOxMeOTf, were examined as initiators for the EtOx polymerization at 90 °C. The initial monomer concentration was kept at 3 M and [M]_0_/[I]_0_ was kept at 50 for the initial kinetic investigation. The monomer conversion was followed by ^1^H NMR spectroscopy and plotted in the common semi-logarithmic kinetic plots (Figure 2).

For the majority of initiators, the kinetic investigation for the EtOx polymerization showed a rather linear pseudo-first-order reaction to high monomer conversions (Figure 2a). The rate of polymerization, as one could expect, depends on the initiator (i.e. leaving group/counterion) used and MeOTf, MeOTs, EtOxMeOTf, and BnBr result in fast initiation. The monomer conversion reached more than 95% after 3 h of incubation (Figure 2a). The ^1^H NMR spectra of the polymer solutions after complete monomer conversion display significant signals at 1.06 ppm and 3.4 ppm attributed to the methyl group in the side chain and PEtOx backbone, respectively. Signals at 1.93–2.34 ppm, 2.60–2.82 ppm, 3.80–4.09 ppm, and 4.94–5.08 ppm are assigned to DLG (Figure 2b, peaks highlighted with asterisks). Also, new signals at 3.05–3.20 ppm, 3.55–3.68 ppm, and 5.38–5.54 ppm point toward products of side reactions (Figure 2b). Interestingly, the signal attributed to the initial methyl group, usually appearing at 3.00 ppm, was not observed in any spectrum, even after the precipitation of the final polymer (Figure S7 in Supporting Information File 1). Signals attributed to the free initiator were also not detected, indicating that all of the initiator had reacted. Initiation with AcCl resulted in a slower monomer conversion and required a reaction time of 24 hours to achieve 90% monomer conversion. The ^1^H NMR spectrum also shows signals attributed to PEtOx and significant signals at 3.52–3.71 ppm attributed to side products as before (Figure 2b, dark red). The use of PropCl as CROP initiator resulted in an extremely low monomer conversion even after 24 hours (9%, Figure 2b, purple). The decrease in the polymerization rate after initiation with AcCl and PropCl compared with aforementioned initiators could be explained by the more nucleophilic Cl^−^ counterion and formation of covalent species [20]. Signals in the ^1^H NMR spectra that can be attributed to DLG complicate the analysis of the data obtained, while the peaks attributed to the terminal oxazolinium proton or formation of termination groups by the covalent mechanism overlap with the solvent or polymer backbone signals (Figure 2b). Interestingly, the SEC traces remain monomodal (Figure S8 in Supporting Information File 1). In addition, the coloration of the polymerization mixture observed earlier is still observable, indicating undesirable side reactions, presumably with the solvent.

**Figure 3:**
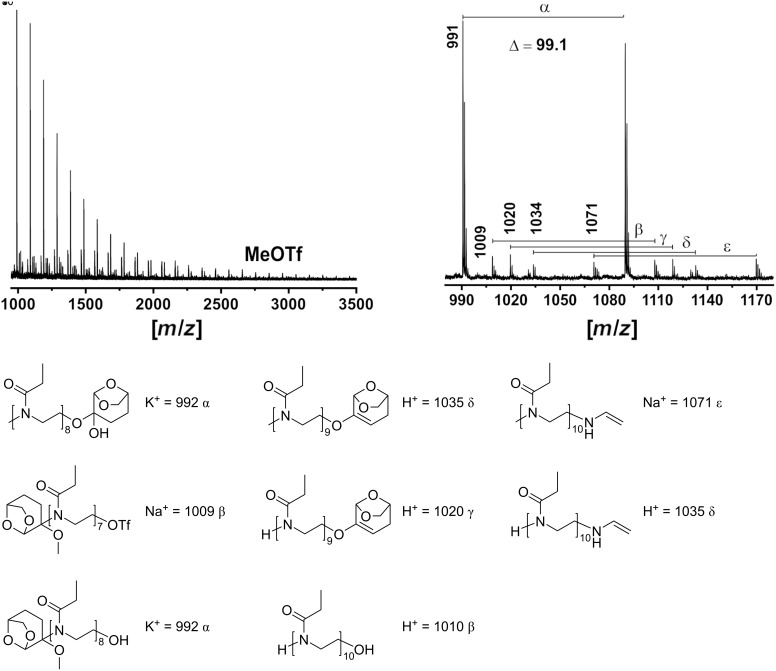
MALDI-TOF mass spectrometry analysis of the poly(2-ethyl-2-oxazoline) initiated with methyl triflate in DLG at 90 °C.

**Figure 4:**
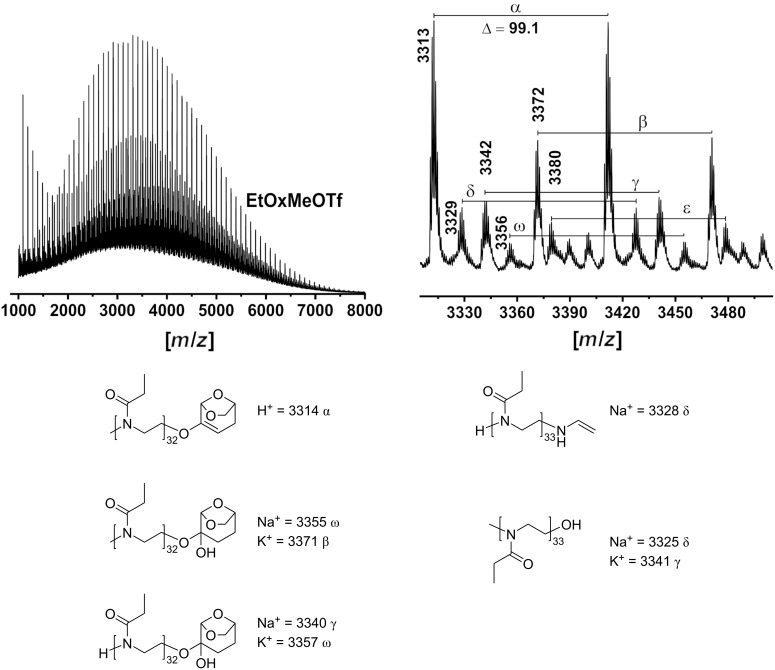
MALDI-TOF mass spectrometry analysis of the poly(2-ethyl-2-oxazoline) initiated with 2-ethyl-3-methyl-2-oxazolinium triflate in DLG at 90 °C.

The products obtained after precipitation from cold Et_2_O and lyophilization were analyzed by MALDI-TOF mass spectrometry (Figure 3, Figure 4, and Figures S9–S11 in Supporting Information File 1).

**Figure 5:**
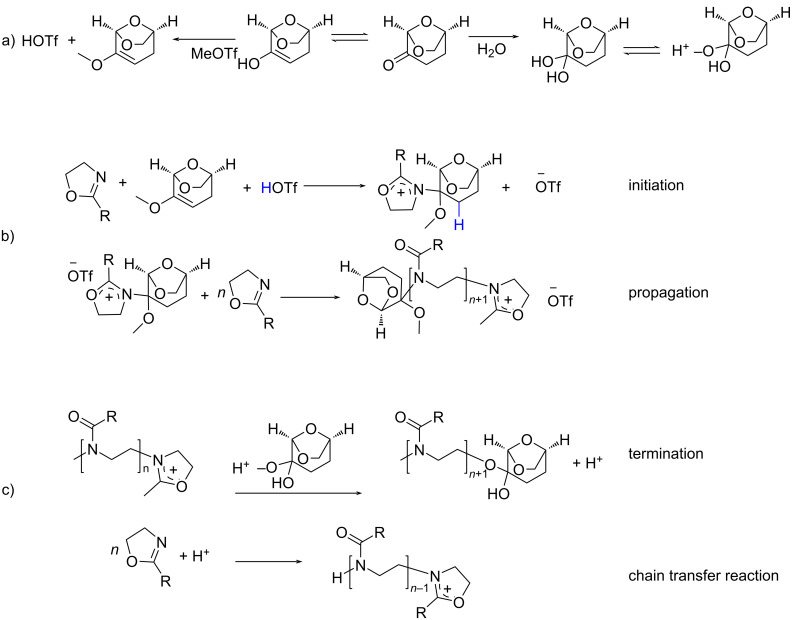
Schematic illustration of the side reactions that can occur during the polymerization of 2-alkyl-2-oxazoline in DLG.

The resulting MALDI-TOF mass spectra clearly confirm the presence of the desired polymer. The mass difference (Δ*m*/*z*) between individual signal distributions differs by 99 g/mol, corresponding to the molar mass of the EtOx repeating unit. The PEtOx initiated with MeOTf, MeOTs, and AcCl did not show a discernible or a narrow molar mass distribution, and the molar mass of the resulting polymers is much lower than expected from the [M]_0_/[I]_0_ = 50 ratio. It is important to note that the main molar mass distribution cannot be attributed to PEtOx chains initiated by methyl or acyl ions and terminated by the OH group, as would be expected if no side reactions occurred. The observed basic molar mass distribution, exemplified by the most intense peak (*m*/*z* = 991) (α), can be attributed to PEtOx chains that are terminated by a molecule of DLG diol with a potassium ion doping (Figure 3). However, α-distribution can also be attributed to DLG-initiated PEtOx. The presence of signals attributed to a polymer with a solvent fragment/derivative and covalently bonded counterion (OTf^−^) species bearing sodium ion doping (β) can also be assigned. This distribution (β) could also be assigned to PEtOx, which is generated by a chain-transfer reaction with H^+^ (Figure 3). To explain these observations, we suggest a set of undesired reactions which interfere with the cationic ring-opening polymerization of 2-oxazolines (Figure 5). It has been reported that DLG can undergo keto–enol tautomerism and form enols or can participate in nucleophilic addition reactions [49]. Furthermore, it can react with water to its hydrated form, a geminal diol (Figure 5a). We suggest that the solvent reacts with methyl triflate to form triflic acid and methylated DLG (Figure 5a). The strong acid triflic acid then can initate the polymerization with a proton [46]. Alternatively, the triflic acid could react potentially together with DLG with a monomer, leading to a DLG-initiated polymer chain (Figure 5b). Alternatively, DLG can, after reaction with water, react as terminating reagent, yet again releasing a proton which can initiate another polymer chain, essentially finishing the chain transfer (Figure 5c). These chain-transfer reactions certainly explain the absence of the methyl initiator group in the NMR spectra of the products [45,50] and are strongly supported by our results from mass spectrometry (Figure 3, Figure 4 and Figures S9–S11 in Supporting Information File 1). However, the lower intensity molar mass distribution (β) can also be attributed to PEtOx produced by the chain-transfer reaction with OH-termini carrying doping proton ions, which is probably a simpler explanation.

The peak at *m*/*z* = 1020 (γ) can be attributed to the desired methyl-initiated PEtOx with DLG-ω-termini bearing proton ion doping. The other molecular weight distributions (ε) and (δ) of low intensity can be attributed to a methyl-initiated and proton-initiated PEtOx formed after termination at position C2 with the final fragmentation of the formed ester group during the MALDI-TOF MS assay carrying Na^+^ and H^+^ ions, respectively. It is well known that water and potassium hydroxide tend to terminate at the 2-position. This results in the formation of POx containing a secondary amine and a cleavable ester terminal group [49]. Subsequent dehydration under conditions of MALDI-TOF mass spectrometry might lead to a dehydration, although this is speculative at this point.

Initiation with MeOTs or AcCl yields qualitatively similar results. The major molecular weight distribution (α) could be assigned to PEtOx with a solvent molecule as a terminating or initiating moiety bearing a potassium ion (Figures S9 and S11 in Supporting Information File 1). Signals at *m*/*z* = 1009 (γ) and 1038 (δ) could also be attributed to PEtOx carrying counter ion termini after initiation with MeOTs and AcCl, respectively.

The mass spectrometric results obtained after initiation with EtOxMeOTf and BnBr were more in line with the expectations (Figure 4 and Figure S10 in Supporting Information File 1, respectively). Utilizing EtOxMeOTf, i.e., a separately synthesized salt of the initiator or propagating species, avoids strong alkylating reagents, which seems to prevent side reactions to some extent. However, a molar mass distribution at lower *m*/*z* is still observed. The main distribution with a peak at *m*/*z* = 3314 could be attributed to PEtOx terminated with a dihydrolevoglucosenone derivative bearing the initiating methyl group and H^+^ (α) ionization. Additional distributions at lower intensity observed peaking at *m*/*z* = 3371 (β) and *m*/*z* = 3355 (ω) could be attributed to methyl-initiated PEtOx with a DLG-diol terminating group carrying potassium and sodium ion doping, respectively. Signals at *m*/*z* = 3325 and *m*/*z* = 3341 could be attributed to methyl-initiated PEtOx with OH moieties carrying Na^+^ (δ) and K^+^ (γ), respectively. We also cannot exclude the possibility that these signals resulted from PEtOx obtained via a chain-transfer reaction containing an NHCH=CH_2_ end group with Na^+^ (δ). The *M*_n_ of the resulting polymers remains lower than expected from the [M]_0_/[I]_0_ ratio.

As observed in MALDI-TOF MS, the primary molar mass distribution of the resulting polymer after BnBr initiation can be attributed to benzyl-initiated PEtOx with Br-termini carrying Na^+^ (α) ion doping. Interestingly, no signals attributed to the initiator were observed in the resulting ^1^H NMR spectra. Signals that could be attributed to PEtOx terminated by water, carrying sodium (β) or terminated by DLG derivatives with K^+^ (γ) or Na^+^ (δ) ion doping could also be observed (Figure S10 in Supporting Information File 1). It is important to note that fragmentation during analysis cannot be excluded. The SEC traces of the resulting PEtOx after initiation with BnBr show monomodality and a relatively broad molar mass distribution (*Ð* ≈ 1.65) (Supporting Information File 1, Figure S8, green), while the polymer obtained with EtOxMeOTf has a higher *M*_n_ and a more narrow molar mass distribution (*Ð* ≈ 1.10) (Figure S8, blue).

### Polymerization of 2-ethyl-2-oxazoline with MeOTf, MeOTs and EtOxMeOTf as an initiator at 60 °C

**Figure 6:**
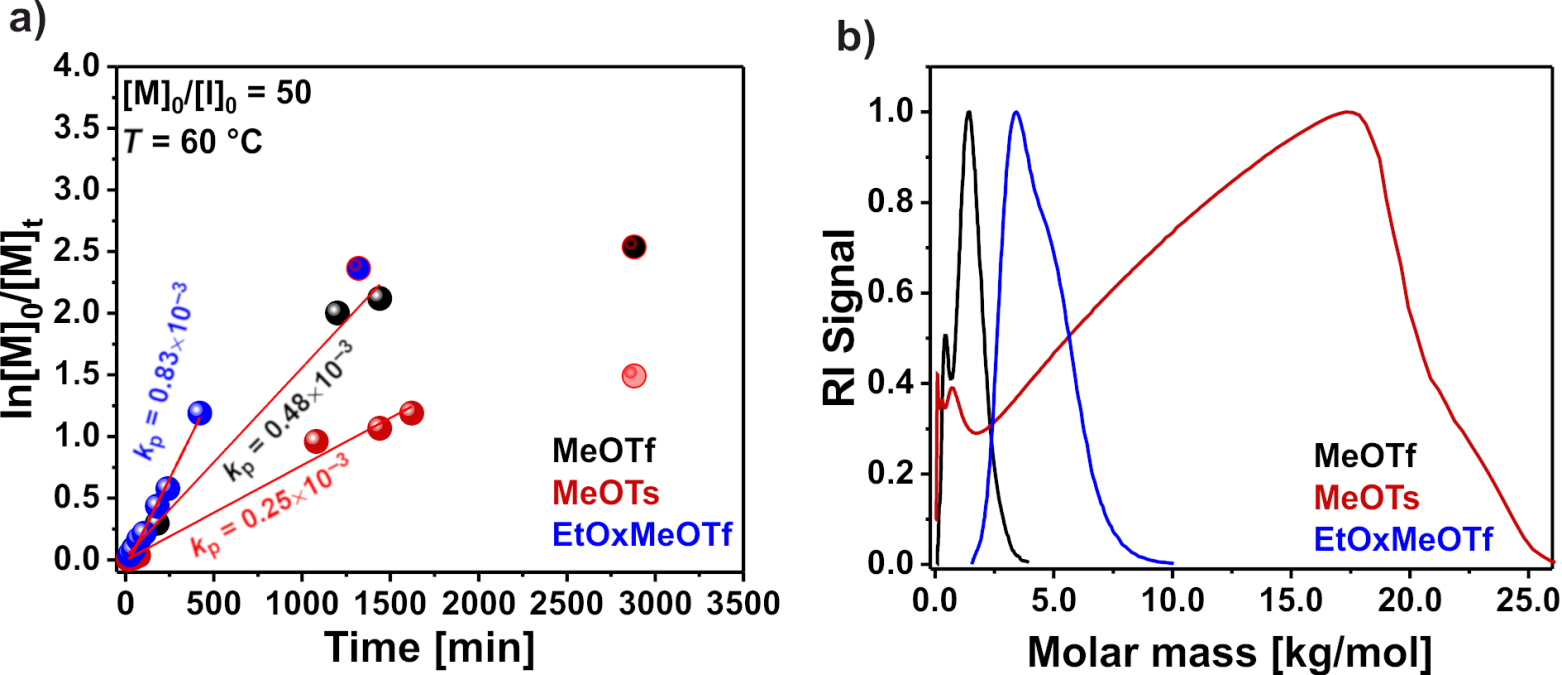
Investigation of 2-ethyl-2-oxazoline polymerization in DLG at 60 °C initiated with MeOTf (black), MeOTs (red), and EtOxMeOTf oxazolinium salt (blue). (a) First-order kinetic plot for the 2-ethyl-2-oxazoline ring-opening polymerization. Circles with red fringes were excluded during the linear fit. (b) HFIP SEC traces of the resulting poly(2-ethyl-2-oxazoline)s obtained at 60 °C.

To potentially further reduce side reactions, we considered lower polymerization temperatures. However, the decrease in the reaction temperature to 60 °C did not show significant improvements (Figure 6). The *k*_p_ after initiation with MeOTf and EtOxMeOTf decreased considerably, from 6.7 × 10^−3^ Lmol^−1^s^−1^ and 6.6 × 10^−3^ Lmol^−1^s^−1^ (during incubation at 90 °C, Figure 2a) to 0.5 × 10^−3^ Lmol^−1^s^−1^ and 0.8 × 10^−3^ Lmol^−1^s^−1^, respectively (Figure 6a). For MeOTs, a decrease of *k*_p_ from 4.4 × 10^−3^ Lmol^−1^s^−1^ (Figure 2a) to 0.3 × 10^−3^ Lmol^−1^s^−1^ (Figure 6a). The kinetic study also showed that monomer consumption initially follows a linear pseudo-first order, but levels off after some time, indicating that the polymerization is slowly terminated, albeit only at high monomer conversion (Figure 6a). The SEC traces of the resulting polymers show a significant difference in the resulting molar mass depending on the used initiator. The molar mass of the PEtOx obtained with MeOTf and MeOTs at 90 °C is much lower (*M*_n_ = 0.82 and 1.37 kg/mol) than expected from [M]_0_/[I]_0_ ratio (5.0 kg/mol) with a broad molar mass distribution (*Ð* = 1.55 and 1.38, respectively) (Figure S8 in Supporting Information File 1). The SEC trace of the PEtOx obtained at 60 °C and initiated with MeOTs is bimodal, and the *M*_n_ is lower than expected (0.51 vs 5.0 kg/mol, Figure 6b, red). After initiation with MeOTf, the resulting *M*_n_ remained much lower than expected from the [M]_0_/[I]_0_ ratio with the presence of low-molecular weight products (Figure 6b, black).

The resulting molar mass after initiation with EtOxMeOTf is closer to what is expected from [M]_0_/[I]_0_ ratio (Figure 6b, blue) and *Ð* remained below 1.2. ^1^H NMR analysis of the final product after precipitation and freeze-drying confirmed the presence of signals attributed to PEtOx, whereas the additional signals detected in the obtained spectra could not be attributed to the monomer or solvent used, nor to the final product after the reaction of the solvent with the initiator.

In addition, EtOx polymerization with EtOxMeOTf as an initiator was also performed at 35 °C. As expected, at this low temperature, the propagation is much slower. Continuous monomer conversion was observed during the 72 hour incubation, but afterwards no significant monomer conversion was observed even after 9 days. The SEC trace for the final polymer after precipitation obtained at 35 °C is bimodal, and the *M*_n_ value is much lower than expected, which is unsurprising considering incomplete monomer consumption. The ^1^H NMR spectra showed the presence of unreacted monomer as well as new signals due to side reactions (Supporting Information File 1, Figure S12). Signals at 1.45–1.75 ppm, 3.21–3.60 ppm, 3.77 ppm, 3.85 ppm, 4.19 ppm, and 4.48 ppm attributable to the product obtained after the interaction of initiator and solvent were not observed in the resulting NMR spectra. However, lowering the polymerization temperature definitely does not lead to improvement.

Finally, a temperature of 120 °C was applied to obtain the PEtOx with [M]_0_/[I]_0_ = 100 repeat units. After only 60 min of incubation, no monomer signals were observed in the NMR spectra, while signals attributed to the desired PEtOx were observed. However, the *M*_n_ of the resulting polymer was again lower than expected (Figure S12 in Supporting Information File 1). Therefore, also higher temperatures do not seem to be beneficial.

### Extending control over the polymerization of 2-ethyl-2-oxazoline with EtOxMeOTf as an initiator

**Figure 7:**
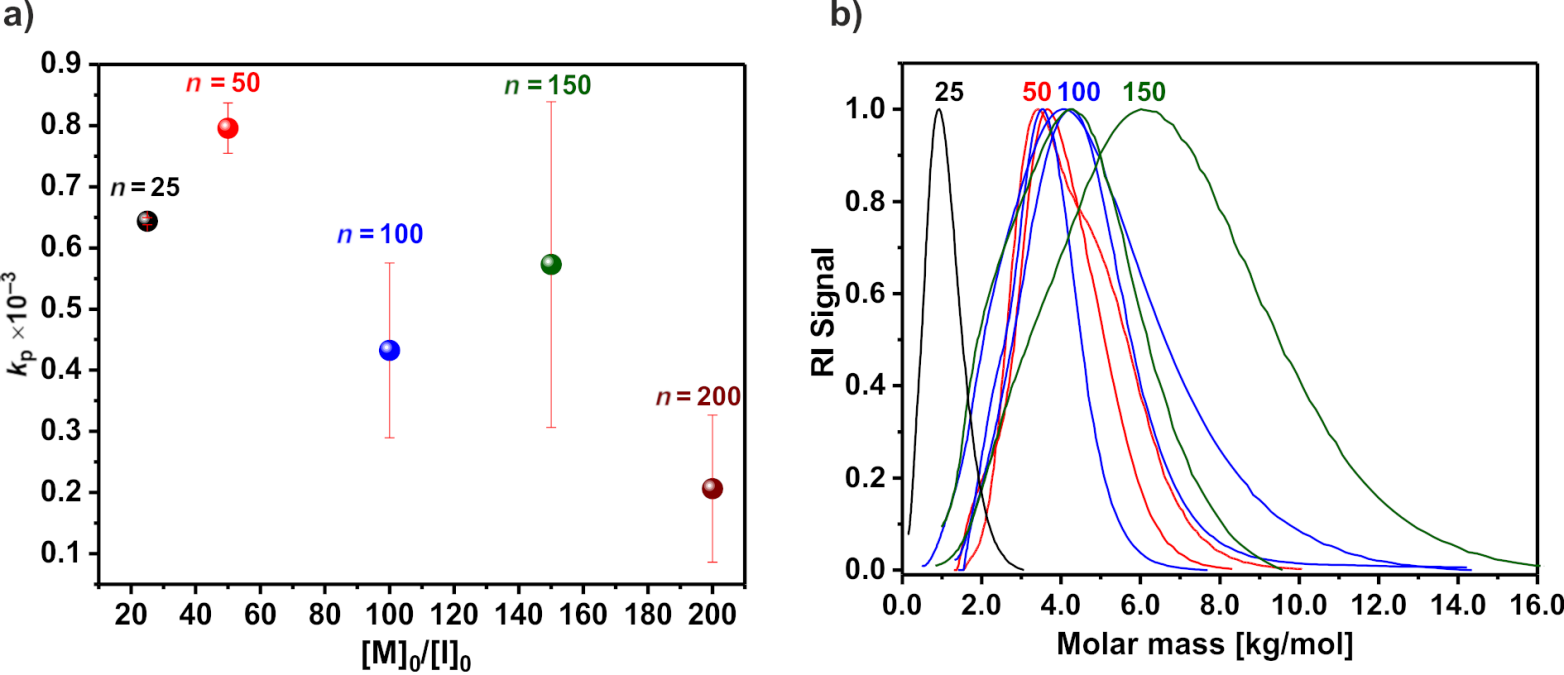
Investigation of 2-ethyl-2-oxazoline polymerization initiated with EtOxMeOTf at different monomer/initiator ratios. (a) Dependence of the apparent polymerization constant on the chain length, calculated from the first-order kinetic plot for the cationic ring-opening polymerization of 2-ethyl-2-oxazoline initiated by EtOxMeOTf in DLG at 60 °C and (b) HFIP SEC traces of the resulting poly(2-ethyl-2-oxazoline)s.

The data obtained after using different polymerization temperatures and initiators showed that the most satisfactory results were obtained at 60 °C and with EtOxMeOTf as initiator. Application of these conditions resulted in a fast monomer conversion rate and the molar mass correlated well with the expected data. Thus, it was decided to perform EtOx polymerization with initial [M]_0_/[I]_0_ = 20–200 (Figure 7) [51].

The plots of monomer conversion versus time for [M]_0_/[I]_0_ = 20 and 50 show a linear pseudo first-order kinetics, indicating a constant number of propagating species during polymerization. The increase of the [M]_0_/[I]_0_ ratio up to 200 leads to increased reaction time and, unexpectedly, a decreased apparent polymerization rate (Figure 7a). A high monomer conversion for PEtOx with higher targeted molar mass was not reached. In addition, the *M*_n_ of the polymers with a lower targeted molar mass correlates better compared to PEtOx with a higher targeted molar mass (Figure 7b). For all polymers *Ð* ≤ 1.4 were obtained and the elugrams were mostly monomodal with some having clear shoulders. The molar mass for PEtOx with [M]_0_/[I]_0_ ≥ 100 was lower than expected. In the case of [M]_0_/[I]_0_ = 200, no polymer was obtained during work-up (precipitation). However, NMR spectroscopy suggested that in the early stages of the reaction, some monomer conversion and polymerization might have taken place but overall conversion was low. This may mean that the initiator reacts preferably with the solvent molecules with little or no further propagation. Poor control of the polymerization process, different apparent *k*_p_ values combined with a wide molar mass distribution and low molecular weight indicate that polymerization is affected by chain-transfer reactions or other side reactions such as recombination or termination.

At 90 °C, an increase of the monomer concentration up to 5 M leads to a decrease of the polymerization rate (*k*_p_ = 3.1 × 10^−3^ Lmol^−1^s^−1^) compared to 3 M (*k*_p_ = 6.0 × 10^−3^ Lmol^−1^s^−1^) (Figure S13 in Supporting Information File 1). Also for [M]_0_ = 0.5 M a significant difference in the *k*_p_ value (*k*_p_ = 2.7 × 10^−3^ Lmol^−1^s^−1^) was observed, with only 70% of the monomer converted after 26 h of polymerization. The SEC traces of the resulting polymer are mostly monomodal but with a lower molar mass than expected. Also no significant difference in *k*_p_ (0.6 × 10^−3^ Lmol^−1^s^−1^ and 0.5 × 10^−3^ Lmol^−1^s^−1^, respectively) was observed after reducing the polymerization temperature to 60 °C when 5 M and 0.5 M EtOx concentrations were applied (Figure S13 in Supporting Information File 1). At 0.5 M, only 63% of EtOx were converted after 24 h. Interestingly, no significant peaks indicating PEtOx formation were detected in the ^1^H NMR spectra during the first 3.0 h of incubation, suggesting a lag time. The SEC traces remain mostly monomodal with a narrow molar mass distribution (Figure S13 in Supporting Information File 1).

### Polymerization and copolymerization of 2-butyl-2-oxazoline

As mentioned, POx are desirable polymers for biomedicine. Under the right conditions, the living nature of CROP makes it possible to produce polymers, copolymers, and block copolymers with different chemical compositions and controlled physical and mechanical properties. The results obtained after EtOx polymerization in DLG indicated poor control of the polymerization process and the potential presence of undesirable terminations and some other side reactions. Nevertheless, we decided to attempt a block copolymerization of BuOx and EtOx to investigate the living nature of the reaction (Figure S14 in Supporting Information File 1).

It is well established that 2-oxazolines with a longer substituent polymerize more slowly than those with a shorter alkyl group. The polymerization of BuOx was investigated before the block copolymerization. BuOx polymerization was initiated by MeOTf and EtOxMeOTf at 90 °C, and fast monomer consumption was observed. The ^1^H NMR spectrum of the polymerization mixture of BuOx with EtOxMeOTf as initiator shows the presence of signals related to the desired PBuOx after incubation for 7.0 h (Figure S14, Supporting Information File 1). The SEC trace of the final polymer appeared bimodal (*Ð* = 2.20) with a lower molar mass (*M*_n_ = 0.49 vs expected 13.6 kg/mol). The molar mass of the resulting PBuOx initiated with MeOTf was also lower than expected (*M*_n_ = 1.35 vs 4.6 kg/mol) with clear bimodality, but the molar mass distribution was slightly lower (*Ð* = 1.45) compared to PBuOx obtained from EtOxMeOTf (Figure S14, Supporting Information File 1).

POx-based block copolymers are well known to formulate poorly water soluble drugs and Haider et al. recently compared the drug loading capacity of PEtOx_25_-*b*-PBuOx_30_-*b*-PEtOx_25_ and PMeOx_25_-*b*-PBuOx_30_-*b*-PMeOx_25_ [52]. Since the MeOx polymerization did not show positive results but EtOx polymerization did work to some extent, we performed a preliminary test for chain extension and to synthesize triblock copolymers. EtOxMeOTf was used to initiate block copolymerization at 90 °C. The *M*_n_ increases after adding each block, indicating chain extension to some extent (Figure S14 in Supporting Information File 1). The ^1^H NMR spectra confirm the presence of signals attributable to both, PEtOx and PBuOx (Figure S14 in Supporting Information File 1). However, again the *M*_n_ of the final polymer is lower than expected and shows a relatively wide molar mass distribution (*Ð* = 1.36). Also, the shape of the SEC trace of the final polymer is not narrow or symmetrical. The resulting block copolymer did not show successful results in the preparation of the drug formulation, which strongly depends on the molar mass distribution and composition of the polymer. In conclusion and somewhat expectedly, DLG is not suitable for the synthesis of high quality block copolymers under given conditions.

## Conclusion

In conclusion, we report for the first time attempts to use the recently commercialized “green” solvent dihydrolevoglucosenone for the cationic ring-opening polymerization of 2-alkyl-2-oxazolines, namely 2-methyl-, 2-ethyl-, and 2-butyl-2-oxazolines. The effect of temperature, monomer concentration, and initiator type on the resulting polymers was investigated. The polymerization of 2-methyl-2-oxazoline was not satisfactory. Only polymers with a lower molecular mass than expected and a broad molar mass distribution were obtained, presumably due to the high reactivity of the monomers.

In order to polymerize the less reactive 2-ethyl-2-oxazoline, various initiators such as MeOTf, MeOTs, BnBr, PropCl, AcCl, and EtOxMeOTf were then investigated. Using EtOxMeOTf at 60 °C with an initial monomer concentration of 3 M gave the most favorable results. The ^1^H NMR spectra showed signals that are attributable to the desired poly(2-ethyl-2-oxazolines), although the resulting polymers had a rather broad molar mass distribution and a lower molar mass than expected. As would be expected, the polymerization rate increased with increasing temperature, and the polymerization exhibited some aspects of a living character. Unexpectedly, MALDI-TOF MS suggest that the dihydrolevoglucosenone group can be present at the end of the chain of the resulting polymer, suggesting it acts as a chain-transfer agent or terminating agent, which can explain the lower than expected molar masses of the polymer. Moreover, the obtained mass spectra indicated termination to the C2 position. Obtaining higher degrees of polymerization proved difficult to achieve. PEtOx with [M]_0_/[I]_0_ = 150–200 showed a decrease in the resulting molar mass as well as a slight decrease in the polymerization rate with increasing polymer length. Similar to EtOx, BuOx shows rapid monomer consumption (no monomer signals were observed after 3 h of reaction) during polymerization at 90 °C, but the final molar mass remains lower than expected with significant bimodality. An attempt at the synthesis of a PEtOx-*b*-PBuOx-*b*-PEtOx triblock copolymer yielded again a broad molar mass distribution and a lower molar mass than expected by the [M]_0_/[I]_0_ ratio. In summary, even though the CROP of selected 2-oxazolines can be carried out in dihydrolevoglucosenone, it does not appear suitable to obtain well-defined poly(2-alkyl-oxazoline)s.

## Supporting Information

File 1 Additional figures and spectra.
