# Supplementary material for: Investigation of cationic ring-opening polymerization of 2-oxazolines in the “green” solvent dihydrolevoglucosenone
Source: Beilstein J Org Chem. 2023 Feb 28;19:217–30. doi: 10.3762/bjoc.19.21 (PMC9989667; doi:10.3762/bjoc.19.21)
Supplement: File 1 — Additional figures and spectra. [file Beilstein_J_Org_Chem-19-217-s001.pdf]

BEILSTEIN JOURNAL OF ORGANIC CHEMISTRY

Supporting Information

for

Investigation of cationic ring-opening polymerization of 2-oxazolines in the “green” solvent dihydrolevoglucosenone

Solomiia Borova and Robert Luxenhofer

Beilstein J. Org. Chem. **2023,** *19,* 217–230. doi:10.3762/bjoc.19.21

Additional figures and spectra

License and Terms: This is a supporting information file under the terms of the Creative Commons Attribution License (https://creativecommons.org/licenses/by/4.0). Please note that the reuse, redistribution and reproduction in particular requires that the author(s) and source are credited and that individual graphics may be subject to special legal provisions.
The license is subject to the *Beilstein Journal of Organic Chemistry* terms and conditions: (https://www.beilstein-journals.org/bjoc/terms)

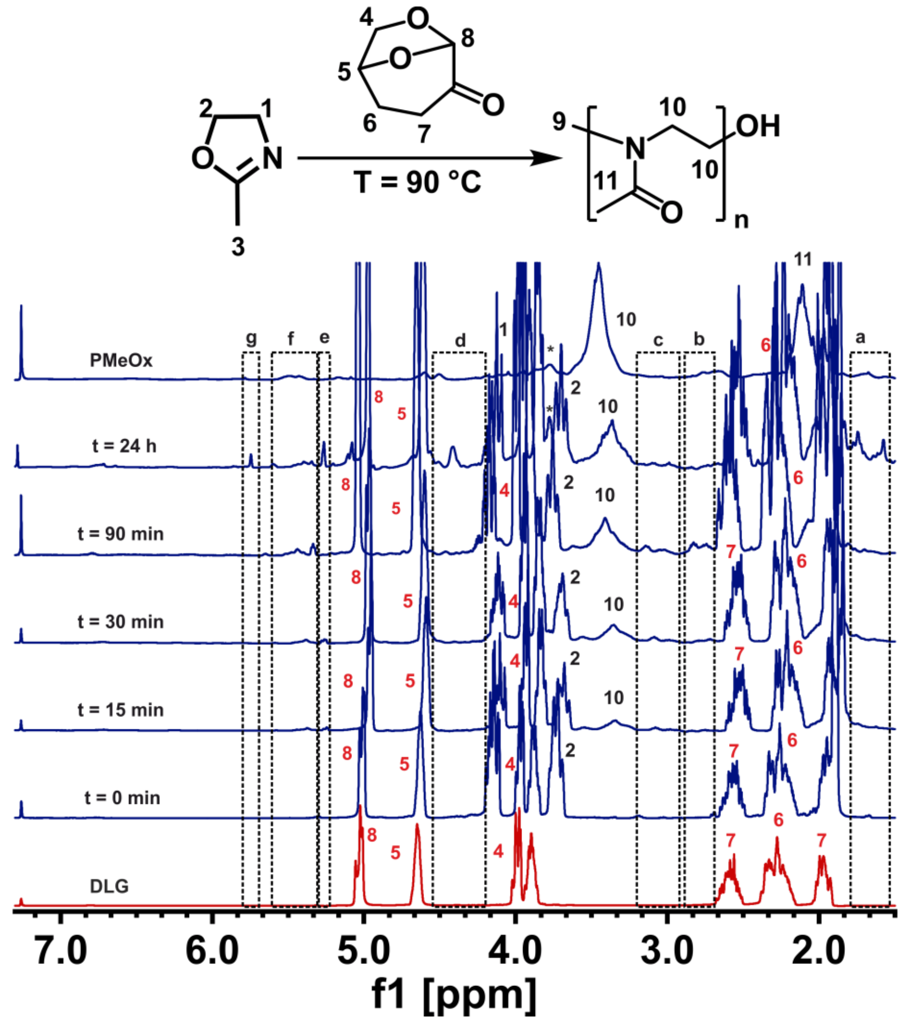

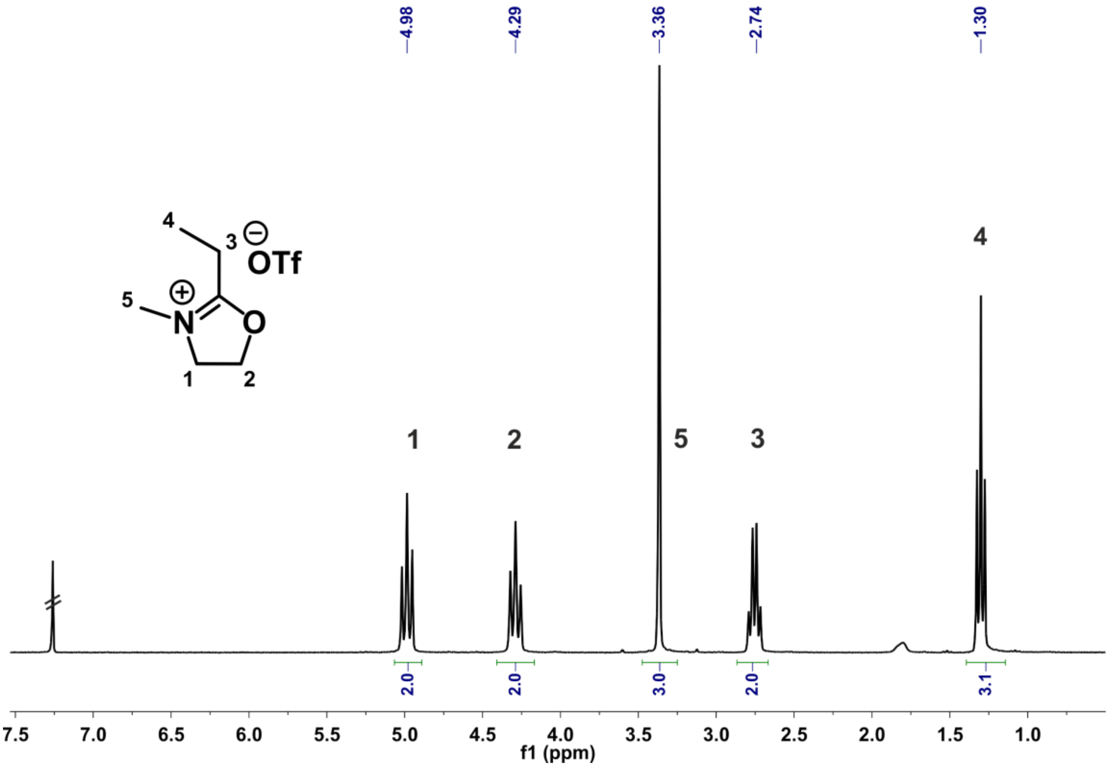

Figure S2. *^{1}H-NMR spectrum (300MHz, $CDCl_3$) of 2-ethyl-3-methyloxazoliniumtriflat after purification.*

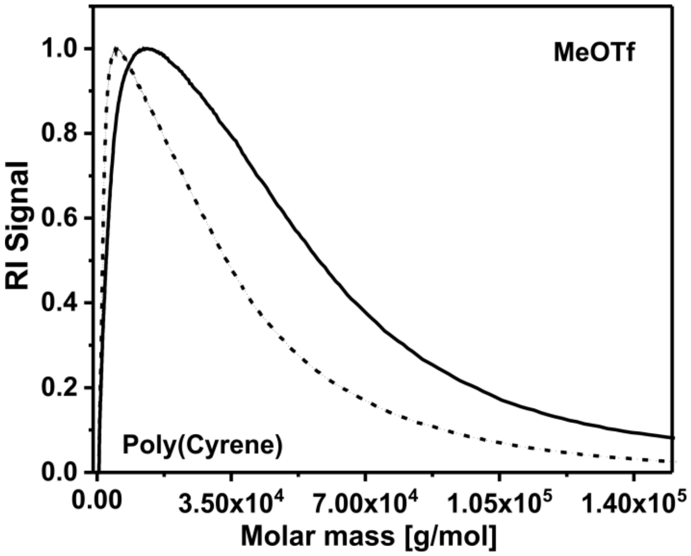

Figure S3. *HFIP SEC traces of the product obtained after incubation of dihydrolevoglucosenone with MeOTf at 90 °C.*

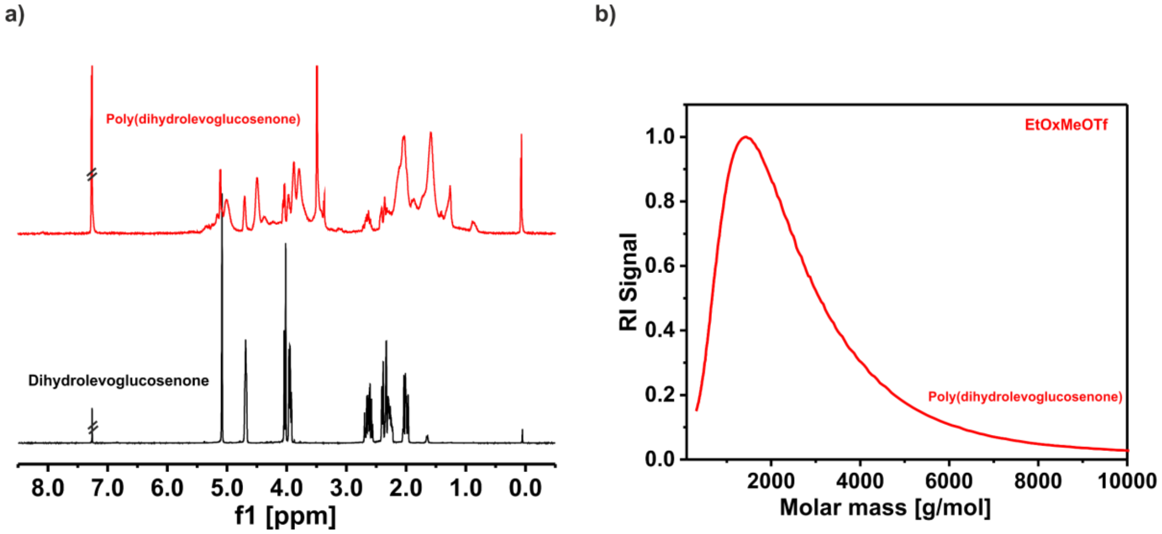

Figure S4. *^{1}H NMR spectra ($CDCl_3$, 300 MHz) obtained after incubation of dihydrolevoglucosenone with EtOxMeOTf at 0°C with worming up to room temperature for 24 h (red). b) SEC trace of the resulting polymer after incubation of dihydrolevoglucosenone with EtOxMeOTf.*

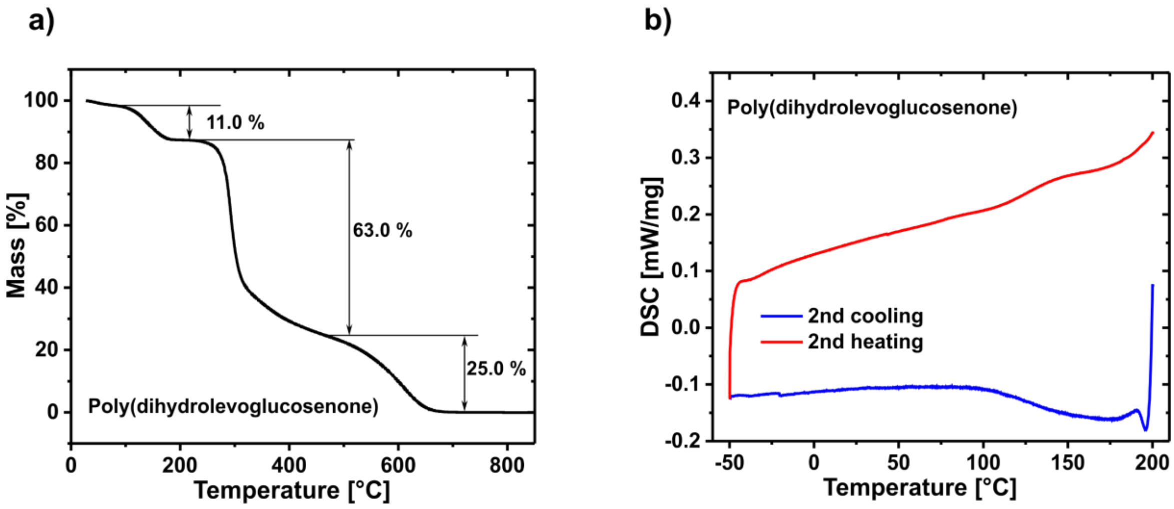

Figure S5. *a) Thermogravimetric analysis (TGA) and b) differential scanning calorimetry (DSC) second heating curve (heating rate: 10 K min^{-1}) of the poly(dihydrolevoglucosenone) obtained after incubation of dihydrolevoglucosenone with MeOTf at 90 °C for 5 minutes.*

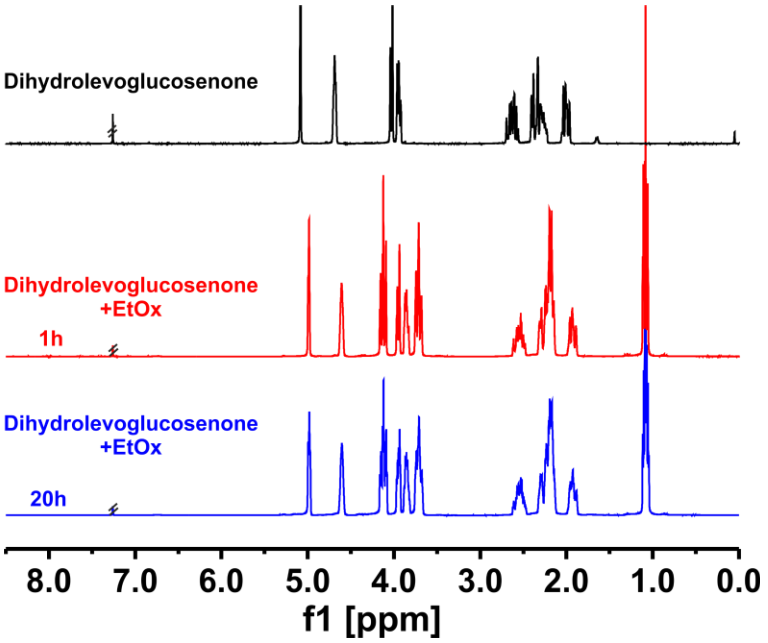

Figure S6*. ^{1}H NMR ($CDCl_3$, 300 MHz) spectra obtained during the incubation of dihydrolevoglucosenone with 2-ethyl-2-oxazoline at 90 °C up to 20 h.*

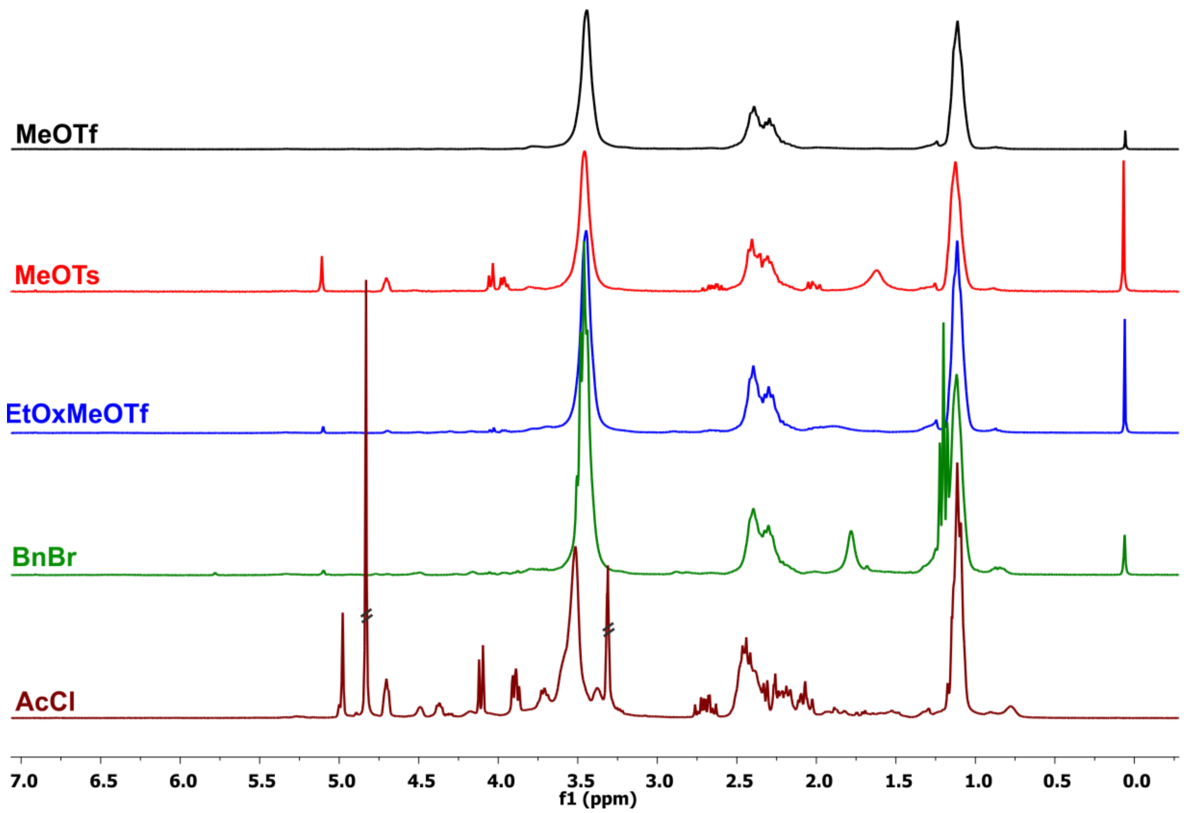

Figure S7. *^{1}H NMR (CDCl3, 300 MHz) spectra of poly(2-ethyl-2-oxazoline) after precipitation using methyl triflate (black), methyl tosylate (red), 2-ethyl-3-methyl-2-oxazoline triflate (blue), benzyl bromide (green) and acyl chloride (dark red) as initiator in dihydrolevoglucosenone.*

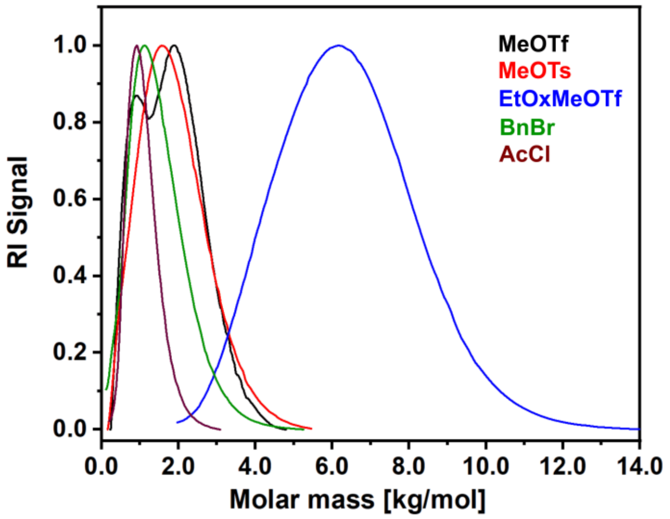

Figure S8. *HFIP SEC traces of the precipitated poly(2-ethyl-2-oxazoline)s obtained in dihydrolevoglucosenone using methyl triflate (black), methyl tosylate (red), 2-ethyl-3-methyl-2-oxazoline triflate (blue), benzyl bromide (green) and acyl chloride (dark red) as initiator.*

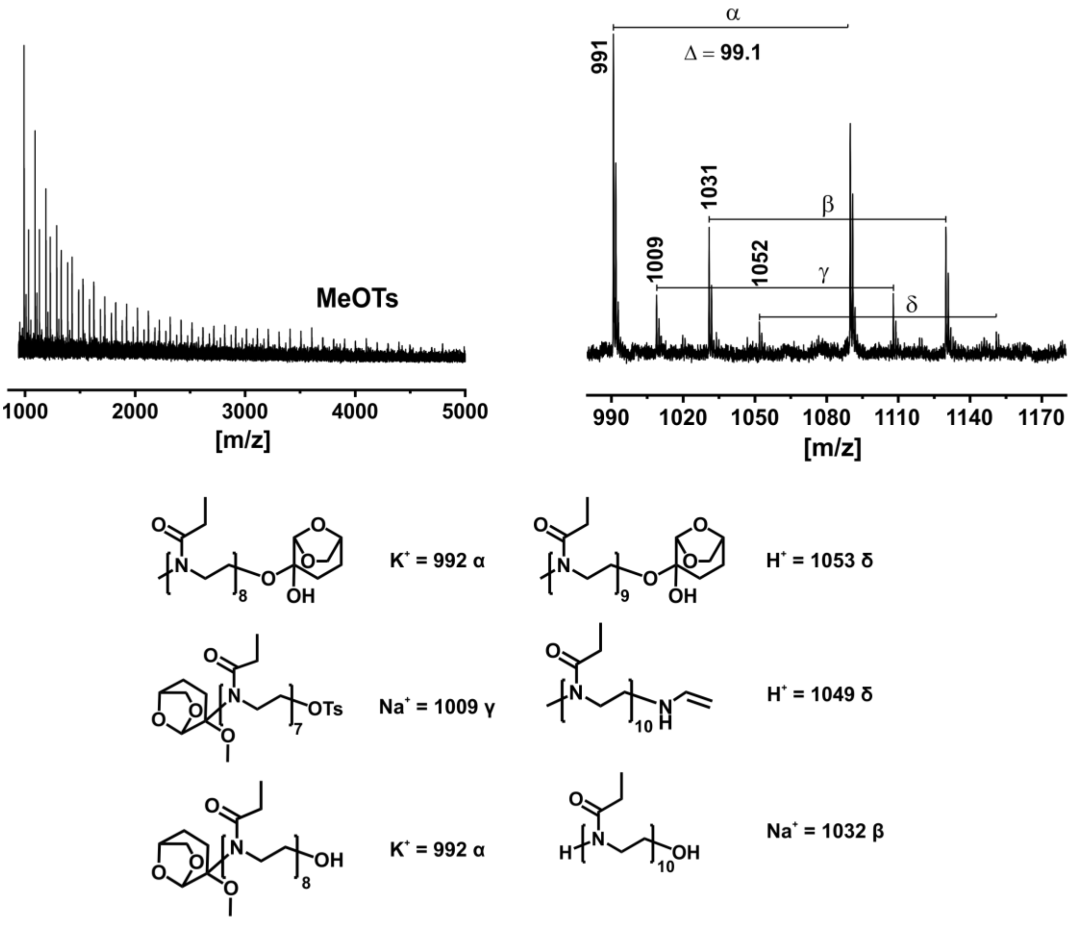

FigureS9. *Maldi-ToF mass spectrometry analysis of the poly(2-ethyl-2-oxazoline) initiated with methyltosylate in dihydrolevoglucosenone at 90 °C.*

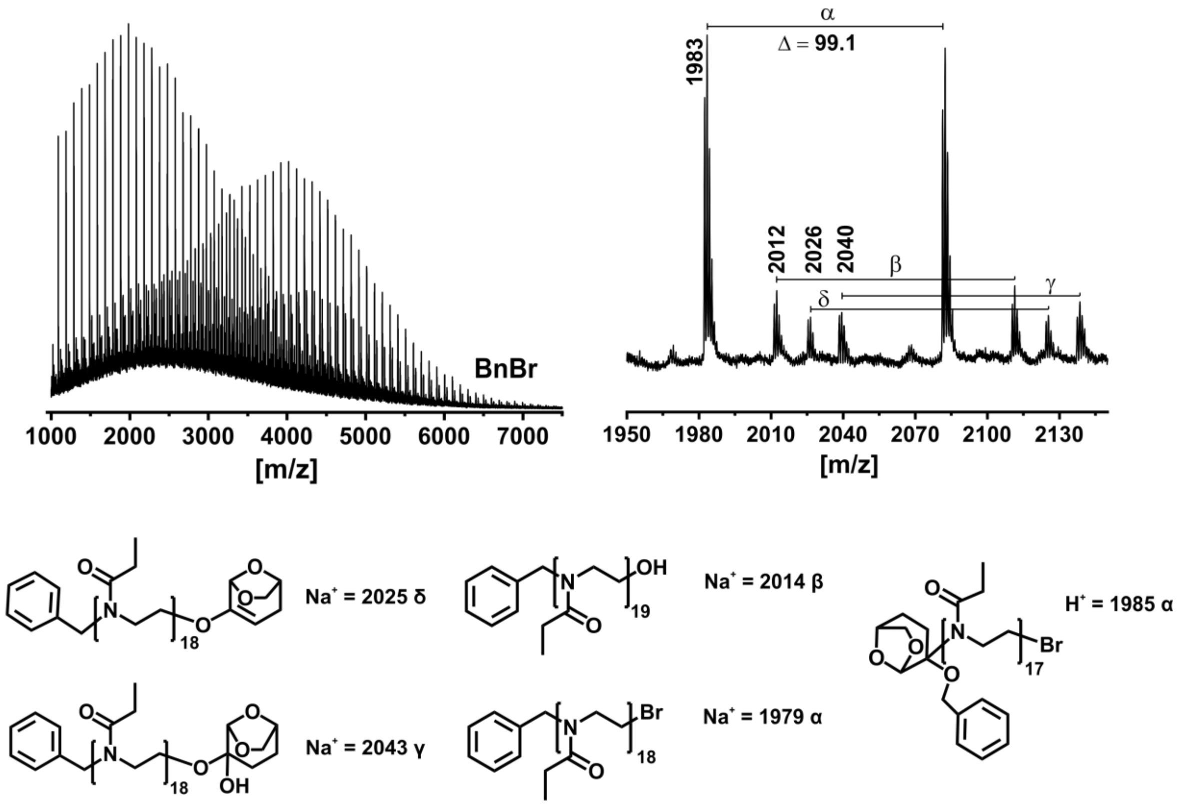

Figure S10. *Maldi-ToF mass spectrometry analysis of the poly(2-ethyl-2-oxazoline) initiated with benzyl bromide in dihydrolevoglucosenone at 90 °C.*

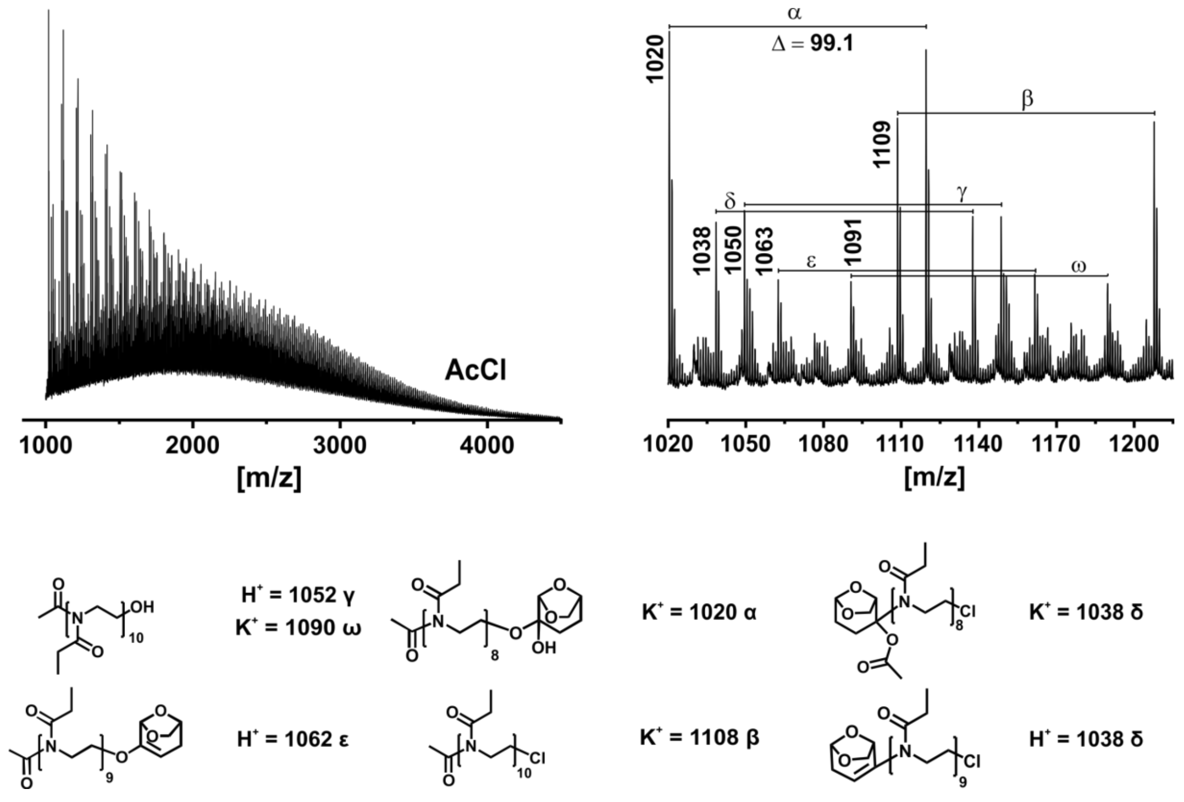

Figure S11. *Maldi-ToF mass spectrometry analysis of the poly(2-ethyl-2-oxazoline) initiated with acetyl chloride in dihydrolevoglucosenone at 90 °C.*

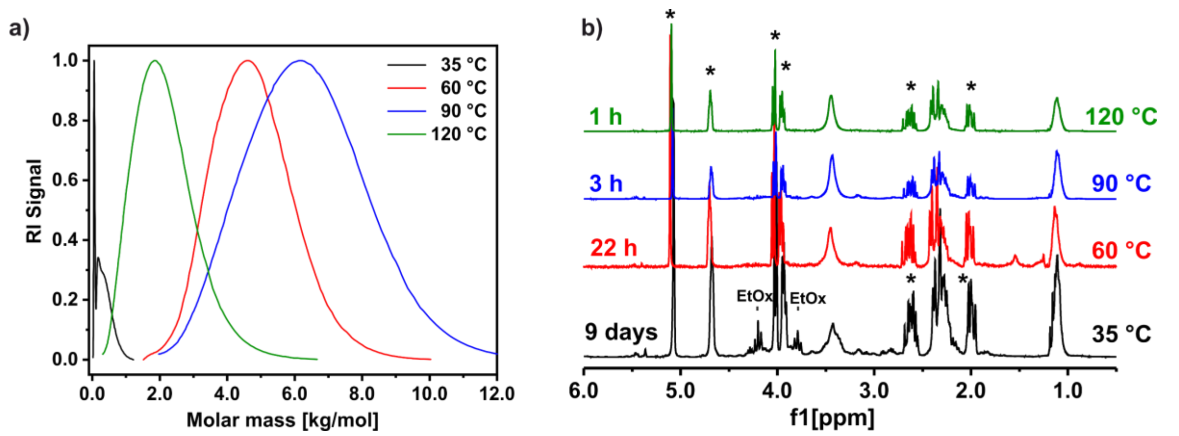

Figure S12. *Investigation of the effect of incubation temperature on 2-ethyl-2-oxazoline polymerization. (a) HFIP SEC traces of the resulting polymers after precipitation and dialysis; (b) ^{1}H NMR (300 MHz, CDCl3) analysis of the resulting poly(2-ethyl-2-oxazoline) polymer solution after complete monomer conversion at 35 °C (black), 60 °C (red), 90 °C (blue) and 120 °C (green). The peaks marked with asterisks are from the residual solvent signals.*

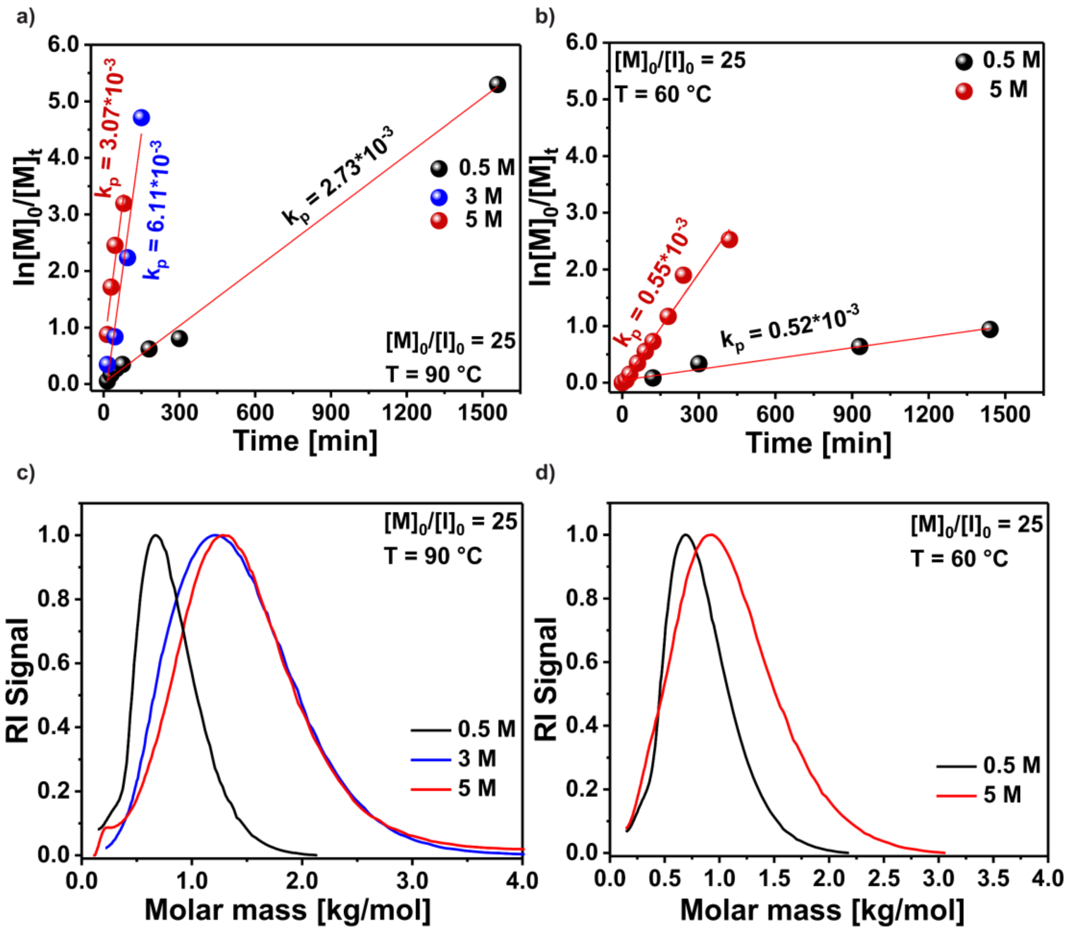

Figure S13. *Kinetic details obtained by NMR for the polymerization with different monomer concentration performed at 90 °C (a) and at 60 °C (b) and the SEC traces of the resulting polymers performed at 90 °C (c) and at 60 °C (d).*

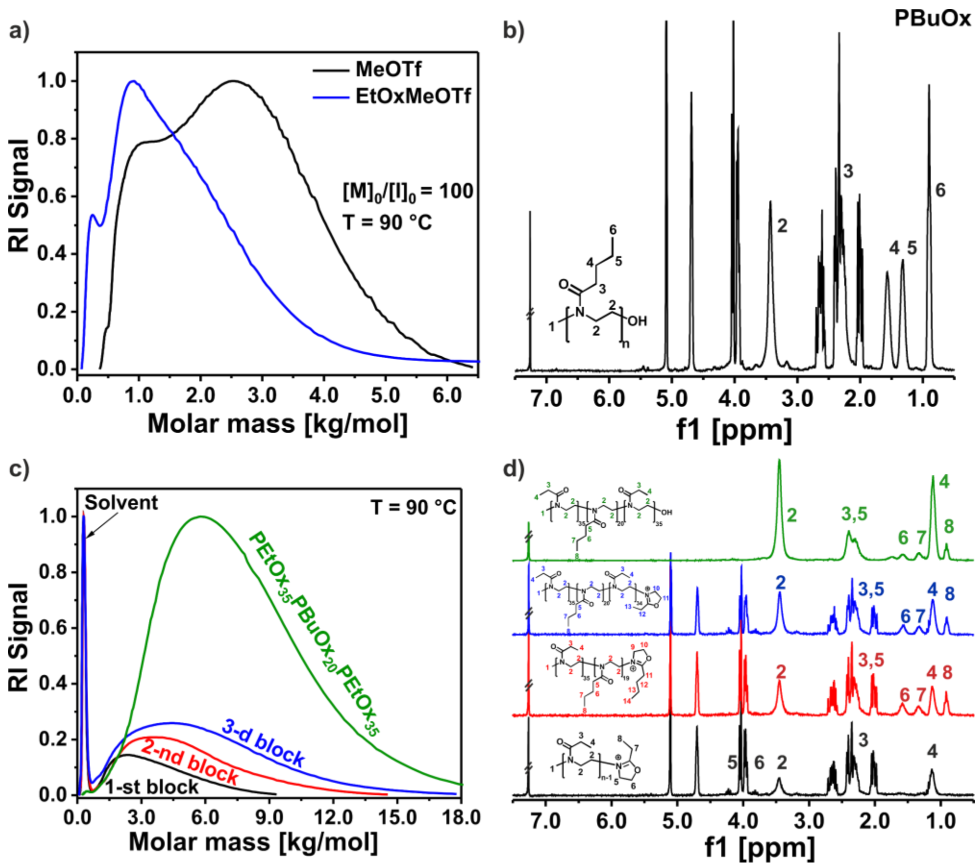

Figure S14. *a) SEC traces of the resulting poly(2-butyl-2-oxatoline) obtained after initiation with MeOTf and EtOxMeOTf at 90 °C in dihydrolevoglucosenone. b) ^{1}H NMR spectra of the resulting polymerization mixture during the BuOx polymerization. c) GPC traces of the resulting block co-polymer of EtOx and BuOx obtained after initiation with EtOxMeOTf at 90 °C in dihydrolevoglucosenone. d) ^{1}H NMR traces of the resulting block co-polymer of EtOx and BuOx obtained after initiation with EtOxMeOTf at 90 °C in dihydrolevoglucosenone.*
